# Connect, collaborate and tailor: a model of community engagement through infographic design during the COVID-19 pandemic

**DOI:** 10.1186/s12889-024-20037-3

**Published:** 2024-09-19

**Authors:** Elizabeth Vernon-Wilson, Moses Tetui, Mathew DeMarco, Kelly Grindrod, Nancy M. Waite

**Affiliations:** 1 School of Pharmacy, University of Waterloo, 200 University Avenue West, Waterloo, ON N2L3G1 Canada; 2 Department of Epidemiology and Global Health, Umeå University, Umeå, Sweden

**Keywords:** Vaccination, Community engagement, Vaccine hesitancy, Vaccine confidence, Vaccine inequity, COVID-19 pandemic, Public health communication

## Abstract

**Background:**

Across the globe, racial and ethnic minorities have been disproportionately affected by COVID-19 with increased risk of infection and burden from disease. Vaccine hesitancy has contributed to variation in vaccine uptake and compromised population-based vaccination programs in many countries. Connect, Collaborate and Tailor (CCT) is a Public Health Agency of Canada funded project to make new connections between public health, healthcare professionals and underserved communities in order to create culturally adapted communication about COVID-19 vaccines. This paper describes the CCT process and outcomes as a community engagement model that identified information gaps and created tailored tools to address misinformation and improve vaccine acceptance.

**Methods:**

Semi-structured interviews with CCT participants were undertaken to evaluate the effectiveness of CCT in identifying and addressing topics of concern to underserved and ethnic minority communities. Interviews also explored CCT participants’ experiences of collaboration through the development of new partnerships between ethnic minority communities, public health and academic researchers, and the evolution of co-operation sharing ideas and creating infographics. Thematic analysis was used to produce representative themes. The activities described were aligned with the levels of public engagement described in the IAP2 spectrum (International Association for Public Participation).

**Results:**

Analysis of interviews (*n* = 14) revealed that shared purpose and urgency in responding to the COVID-19 pandemic motivated co-operation among CCT participants. Acknowledgement of past harm, present health, and impact of social inequities on public service access was an essential first step in establishing trust. Creating safe spaces for open dialogue led to successful, iterative cycles of consultation and feedback between participants; a process that not only helped create tailored infographics but also deepened engagement and collaboration. Over time, the infographic material development was increasingly directed by community representatives’ commentary on their groups’ real-time needs and communication preferences. This feedback noticeably guided the choice, style, and presentation of infographic content while also directing dissemination strategies and vaccine confidence building activities.

**Conclusions:**

The CCT process to create COVID-19 vaccine communication materials led to evolving co-operation between groups who had not routinely worked together before; strong community engagement was a key driver of change. Ensuring a respectful environment for open dialogue and visibly using feedback to create information products provided a foundation for building relationships. Finally, our data indicate participants sought reinforcement of close cooperative ties and continued investment in shared responsibility for community partnership-based public health.

**Supplementary Information:**

The online version contains supplementary material available at 10.1186/s12889-024-20037-3.

## Background

In March 2020, the World Health Organisation (WHO) declared a global pandemic as the novel coronavirus, COVID-19, spread rapidly across the world. While every demographic was affected by this global health emergency, health disparities, defined by the Centers for Disease Control and Prevention (CDC) as ‘preventable differences in the burden of disease, injury, violence or opportunities to achieve optimal health that are experienced by socially disadvantaged populations”, have led to soberingly different health outcomes with COVID-19 incidence, prevalence, and mortality. Among developed countries, ethnic minority populations including people with Black, South Asian and Indigenous heritage have been disproportionately impacted by COVID-19 compared to white counterparts despite wide-ranging public health measures [1–5]. Underlying socioeconomic stressors, such as more prevalent multi-occupancy living and employment that increases likelihood of exposure and makes adherence to social isolation policies difficult, may explain the disparities in COVID-19 impact [6–8]. Other aggravating factors include poorer access to healthcare and information on preventing COVID-19 infection including vaccination [9].

The development and approval of COVID-19 vaccines presented a way to combat the pandemic. However, access to vaccines and vaccine hesitancy were highlighted as key barriers to patient uptake and the development of ‘herd immunity’ [10–13]. The challenge of improving access to COVID-19 vaccines has united public health authorities with health care providers and community organisations in efforts to increase their capacity to deliver vaccines and reach into communities.

Vaccine hesitancy, defined by the WHO as ‘delay in acceptance or refusal of vaccines despite availability of vaccination services” [14] has proved to be a more mercurial problem contributing to suboptimal COVID-19 vaccine uptake [15–17]. The decision to vaccinate is complex, context-specific and influenced by a spectrum of factors including attitudes, beliefs, and behaviours of an individual and the social groups they inhabit [18–20]. Vaccine confidence may vary by vaccine type, from community to community, individual to individual and be changeable over time. Understanding, in real-time, factors affecting vaccine confidence and hesitancy at community and individual level is thus an important step towards improving vaccination rates, safeguarding individuals’ and communities’ health, and protecting those who cannot be vaccinated by creating herd immunity conditions. Public engagement activities that promote dialogue between health providers and communities with low vaccine uptake can help develop such understanding and trust [21]. Engagement efforts that seek out common goals and are collectively motivated to improve health outcomes and level power dynamics that disadvantage patients in health care systems can do much to improve vaccine acceptance and uptake [21, 22].

One way in which community engagement delivers benefit is through its unique potential to make health communication relevant and authentic by increasing cultural adaptations and cultural grounding [23]. By working with community knowledge-holders, health messaging can be reframed and aligned better with ethnic minority target audiences’ values, beliefs and traditions [14, 24]. Community engagement can also aid dissemination of health information. Using local, influencers may extend message reach farther by verbalising information and contextualising the message in new, appealing ways such as pro-vaccination music videos and video clips [9, 25, 26]. Enabling trusted messengers within communities and adopting preferred communication channels lends assurance to messaging that government institutions and health officials may lack, a problem magnified during the COVID-19 pandemic and exacerbated by inadequate provision of timely, culturally-adapted information [21, 25–27]While community engagement is credited with helping reach under-resourced communities, engagement nominally covers a spectrum of activities from informing or consulting communities through to involvement, collaboration and shared leadership that leads to increased individual and community capacity. The International Association for Public Participation (IAP2) spectrum provides a useful analytical framework for understanding levels of public engagement and their potential impact [28, 29]. The IAP2 spectrum outlines five progressive levels of public engagement characterised by increasing community influence on decision making. The IAP2 begins with (1) informing people through top-down communication, (2) consulting via surveys, focus groups and town hall meetings, (3) involving through workshops, (4) collaborating through participatory decision making and consensus building, (5) empowering people as decision makers (Appendix 1). Most representations of the IAP2 present a linear sequence, but it can be helpful to consider engagement as a recursive process in which new circumstances require iterations of early phases to prevent “silo” thinking and steadily build co-operation. The purpose of this study is to explore the first-hand experiences of CCT participants co-operating in a community engagement model that aimed to improve COVID-19 vaccine acceptance. The IAP2 framework is used to describe the evolution of community engagement through the activities and relationships that connected people with expertise in delivering vaccination services and multimedia communication with ethnic minority community representatives from the Region of Waterloo, Ontario. Although the importance of patient engagement is receiving increased attention, current health care literature possesses limited description of integrating community engagement within multidisciplinary response teams. By exploring CCT participants’ experiences of working together, this paper describes the CCT process and outcomes as a community engagement model that identified information gaps and created tailored tools to address misinformation and improve vaccine acceptance during a time of crisis.

## Methods

### Study background & approach

The Connect, Collaborate, and Tailor: Multimedia tools to promote vaccine confidence (CCT) project was funded by the Public Health Agency of Canada’s (PHAC) Immunization Partnership Fund. Ethics approval was granted for this work by the University of Waterloo, ON Office for Research Ethics (ORE 43633). The project objective was to create a novel intersectoral coalition capable of delivering innovative multimedia resources, including infographics, to support health care providers (HCP) and communities build confidence in COVID-19 vaccines through provision of targeted and tailored evidence-based information tools. CCT core participants included researchers from the University of Waterloo (School of Pharmacy, Department of Communication Arts), public health professionals from Region of Waterloo Public Health and community leaders of target audiences identified by Region of Waterloo Public Health. Community leaders accepting the opportunity to participate in the CCT included members of the Region’s Equitable Vaccine Working Group (a temporary group drawing on the Region of Waterloo’s Anti-Racism Advisory Working Group and Vaccine Distribution Task Force) and represented faith and cultural communities with connections to Nigeria, Sudan, Somali, Ethiopia, Caribbean, Rohingya and Indigenous Canadians (details in Appendix 2). Academic researchers had expertise in pharmacy practice, innovative vaccination approaches, advancing social equity through public and digital technologies, public health systems and COVID wastewater management research.

**Fig. 1:**
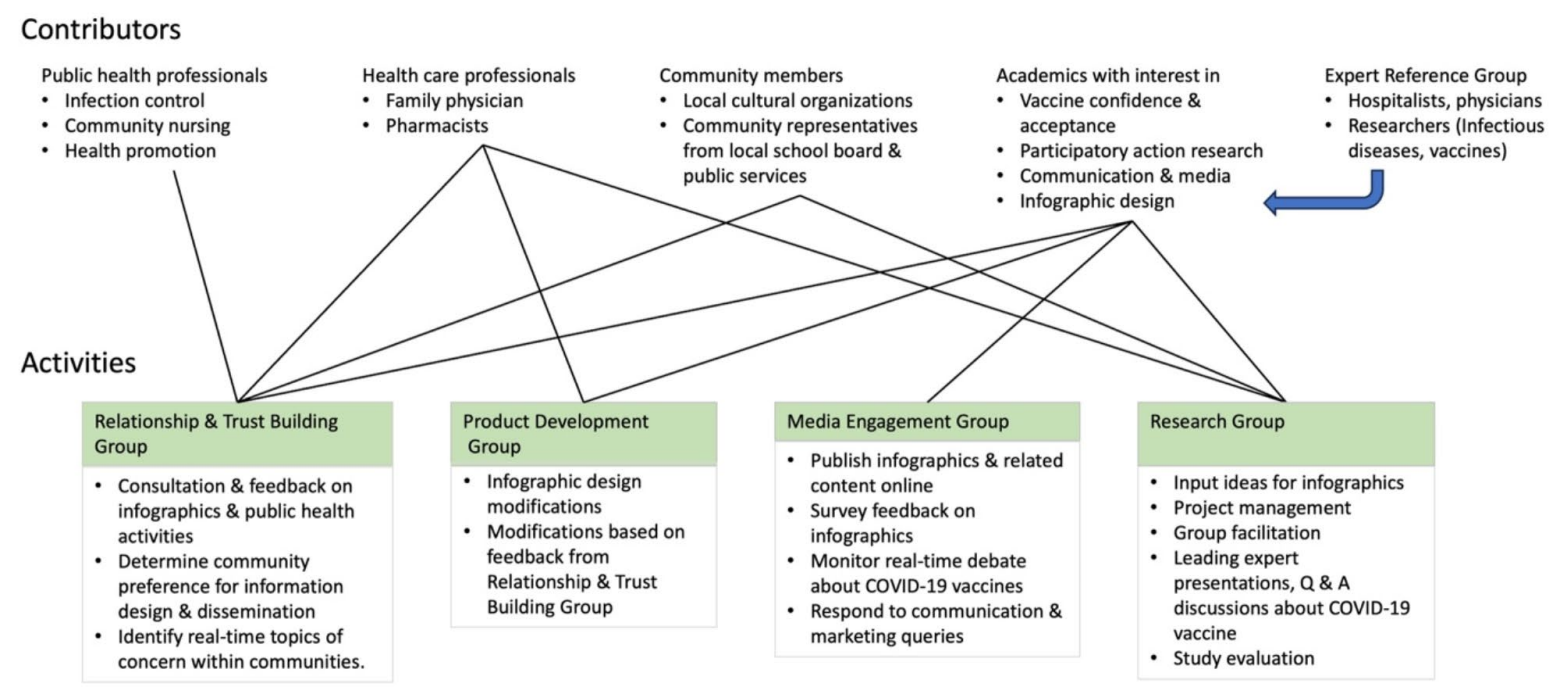
Contributors to and activities of the CCT collaboration working groups

The CCT collaboration began meeting in November 2021, using the Microsoft Teams online meeting platform. Four working groups were subsequently formed to focus on specific activities: the Relationship and Trust Building Working Group, the Product Development Group, the Media Engagement Group and the Research Group (Fig. 1). A fifth Expert Reference Group consisting of physicians and researchers known to CCT leadership team provided additional advice and expertise on COVID-19 treatment and vaccines. This study presents qualitative thematic analysis of project participants’ evaluation interview transcripts.

### Process for creating community-informed infographics

**Fig. 2:**
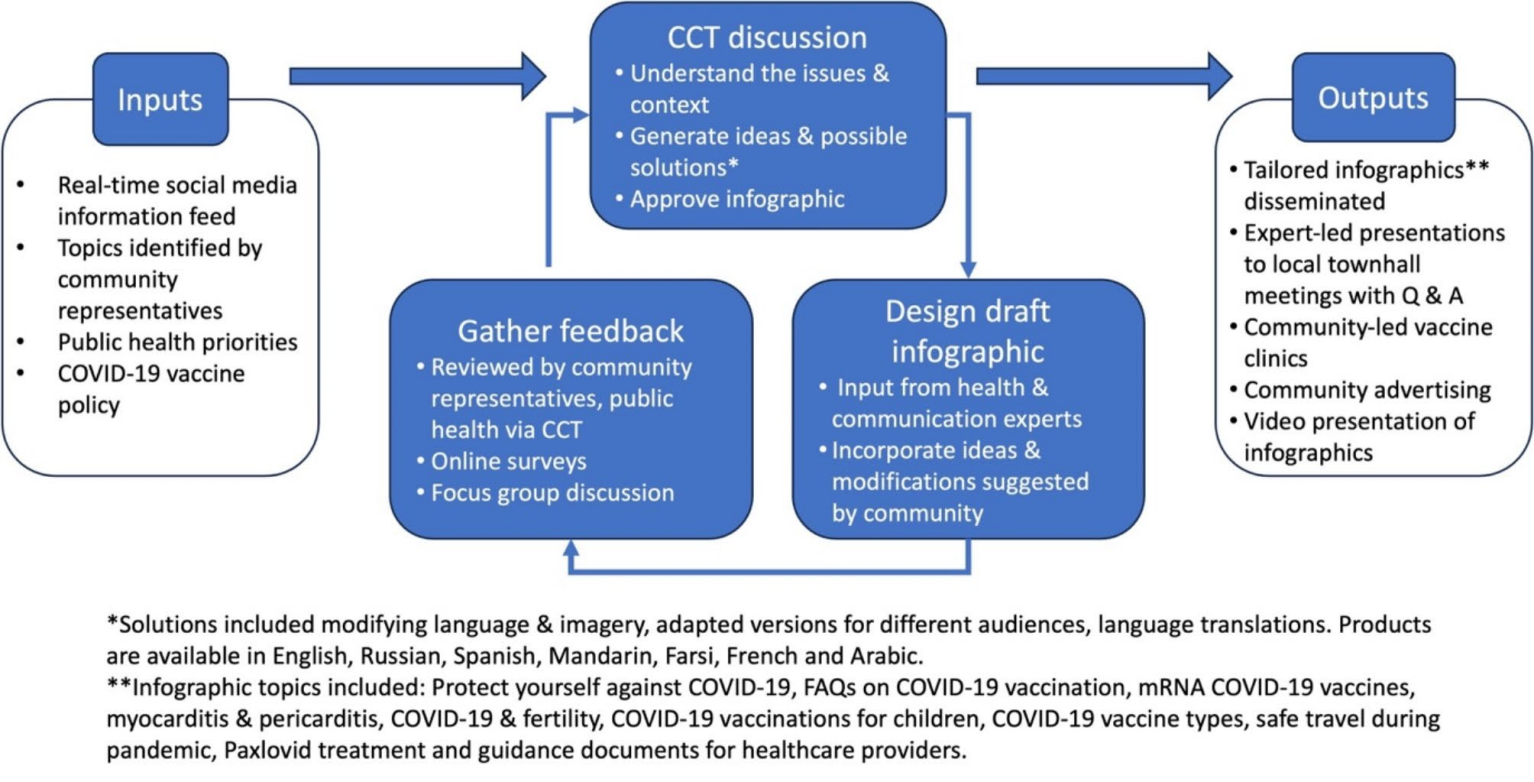
The process for creating community-informed infographics

Topics of interest or concern about COVID-19 infection, vaccines and public health measures were brought forwards for open discussion by the CCT (Fig. 2). Draft infographics were produced by the Product Development Group and reviewed by the Relationship and Trust Building Group to assess whether cultural modifications were desirable for ethnic minority audiences. Additional input was sought through online surveys and focus group discussion. Changes suggested by these routes were used to modify draft infographics, creating multiple versions that prompted further discussion by the Relationship and Trust Building Group. Secondary review and refinement cycles were used to generate improvements as needed. Content approved by the Relationship and Trust Building Group was disseminated as digital and paper products by the Media Engagement Group, the Region of Waterloo Public Health and the University of Waterloo, School of Pharmacy.

### Recruitment of interview participants & consent

The core participants in the CCT collaboration were invited by email to participate in an online interview to assist with study evaluation. Consent to participate in interviews and permission to reproduce quotes anonymously was obtained. Before undertaking interviews, participants were reminded of their right to withdraw consent at any time. Interviews were undertaken in Dec 2022-Jan 2023.

### Interview design & data collection

A semi-structured interview guide informed by PHAC project evaluation criteria was developed using the expertise of the research team. The interview guide focused on (1) the extent to which the CCT model fostered linkages between community, academic and public health partners; (2) the success and challenges of the CCT process; (3) the potential sustainability, scalability, and transferability of the CCT model. While interview questions (Appendix 3) guided discussion, the interviewer was trained to remain flexible and open to emerging ideas about collaboration, developing relationships between communities and public health services and future opportunities for community engagement as participants reflected on their CCT experience. All interviews were undertaken virtually using the online MS Teams platform. On average, interviews lasted 59 min. Interviews were transcribed verbatim prior to analysis.

### Data analysis

Qualitative data were generated from transcripts of recorded semi-structured interviews. Open coding and code organization were supported by use of NVIVO™ software (Version 12.7). An inductive and deductive approach to data analyses was undertaken [30] by authors with differing professional backgrounds. Data analyses were completed in six stages starting with data familiarization through repeated reading of transcripts and preparation of summary notes. Next, initial inductive coding, by identifying significant words and phrases, was undertaken. Codes were grouped where similar meaning or constructs were detected. A second author read all interviews, generated summaries and validated codes and groupings. Code groups were discussed, rearranged and named. Further categorization of groups of codes was undertaken to form sub-themes and higher order themes. The homogeneity and meaning of groups, sub-themes, themes and reflexivity were discussed with other authors, who were part of the CCT Research Group and participated in evaluation interviews.

A basic secondary analysis was performed to map CCT activities to elements of the IAP2 model. In brief, activities described by participants were collated and aligned with IAP2 levels. Fit was confirmed by the wider CCT Research Group and representative examples selected.

## Results

### Participant information

Fourteen participants of the CCT group took part in interviews of whom five were male and nine were female. The CCT group had diverse ethnic and cultural backgrounds; participants identifyed as Caucasian (36%), Black (43%), Asian or Polynesian descent (21%). All contributors had participated in the Relationship and Trust Building Group, in addition four had contributed to the Product Development Group, four to Media Engagement Group, and seven to the Research Group (Table 1). Additional information about interview participants’ partnership affiliations can be found in Appendix 2.

**Table 1:** Roles and affiliations of CCT participants who contributed to evaluation interviews

Interview participant	Primary affiliation/ interest	Relationship/ Trust Building Group	Product Development Group	Media Engagement Group	Research Group
1	Region of Waterloo Public health	x			
2	Physician/ Public health	x	x		x
3	Community representative	x			
4	Academic research -communication	x		x	x
5	Community representative	x			
6	Community representative	x			
7	Academic research -communication	x		x	x
8	Academic-Microbial research	x			x
9	Communication	x	x		
10	Region of Waterloo Public health	x			
11	Region of Waterloo Public health	x			
12	Academic research -vaccine delivery & uptake	x	x	x	x
13	Academic research -public health systems	x	x	x	x
14	Academic research -vaccine delivery & uptake	x			x

Analysis of interview data identified activities undertaken that progressively contributed to meaningful community engagement. Participants stated partnerships and co-operation within the CCT evolved as regular discussions took place between representatives of ethnic minority groups in underserved and marginalized communities, public health, and researchers. This involvement revealed previously unrecognized communication gaps and priorities relating to public health measures, COVID-19 infection and vaccination that could be targeted with information resources. Subsequent collaboration developing infographics, together with iterative feedback discussions, led to tailoring and refinement of both these resources and dissemination processes (Fig. 2). In addition, improving communication links with underserved populations led to other bespoke activities co-produced with community input that promoted COVID-19 vaccination such as reproducing written information in video format, improved promotion of mobile vaccination units and community-led vaccination clinics utilising local venues.

### Mapping to IAP2 model

Activities described by the CCT participants were aligned to the IAP2 model. The CCT actions ranged from IAP2 level 1 (informing) through to level 5 (empowering); examples are shown in Table 2. More references were made to level 1, 2 and 3 activities than level 4 & 5 activities in interviews. These latter, higher-level engagement stages were described as appearing later in the collaboration period. In parallel, interview participants observed relationships between community representatives and public health professionals took time to develop and that higher level engagement activities, as described in IAP2, were facilitated by trusted relationships. As understanding and trust within the CCT group gradually improved, greater participatory decision-making and collaborative efforts became possible and later the norm “*As we went along*,* it became a much more collaborative process*” (CCT11). Participants noted “*the most successful mobile clinics*” when “*there was community input into the organization…the dynamics changed*” (CCT13). When public health provided resources and local people co-ordinated the work, “even the nurses said ‘*OMG! Where did these people come from*?’” (CCT06).

**Table 2:** Examples of IAP2 community engagement level activities undertaken by CCT

IAP2 engagement level	Activity undertaken
Inform	Provide evidence-based information on COVID-19 vaccines in translated forms (basic information on access, availability, priority groups)
Consult	Gather feedback on suitability of content and language used in infographic through surveys (e.g. COVID-19 & fertility - what you need to know)
Involve	Making connections and consistently seeking input to adapt design and content of information tools through CCT groups. Working together to make suitable alternatives for different audiences. Looking for alternatives ways to disseminate information based on community preference (e.g. COVID-19 & fertility - what you need to know; COVID-19 vaccines for children) .
Collaborate	Building robust relationships that enable active enquiry for content ideas from community members. Adopting community preferences and guidance throughout infographic design process. Being led by community views whenever possible. Sharing design, process and outcomes. (e.g. information about travel, vaccination and preventing infection).
Empower	Community-based organisation creates video based on information tool. (promoting COVID-19 vaccination, myth-busting) Community co-ordinates vaccine clinic resourced by public health (Somali community in Waterloo Region).

### Thematic analysis of CCT experience

Thematic analysis of interview data led to the development of three interconnected themes that describe how the processes and output of community engagement and collaboration across the CCT were influenced both positively and negatively by (1) unique pandemic conditions, (2) evolution of infographic development process, (3) wider societal context. These themes are detailed below.

### 1) Unique pandemic conditions shaped collaboration and engagement

Forming an intersectoral coalition during a pandemic, the CCT, brought together people with different cultural and professional backgrounds, lived experiences and expertise who had not routinely interacted or collaborated before. Reflection on the CCT infrastructure and how work was co-ordinated shed light on perceived enablers and constraints to the process of joint working to make informative infographics that could address public concerns about COVID-19 infection, public health measures and vaccinations. Common observations expressed showed that the pandemic environment situated the group’s activities in an unparalleled context.

#### Urgency and shared purpose

Co-operation in the CCT came about through shared vision. Academics with specialities in pharmacy practice, medication communication, graphic design, digital communication, public health professionals and representatives from underserved communities all saw an unprecedented need to improve communication about COVID-19 transmission, infection and vaccination. One academic explained they were motivated to “*have a much closer connection to community*” to increase vaccine acceptance at a time of ‘*crisis*’ (CCT12). Across the collaboration, interviews revealed how people saw themselves rising to this challenge; responding to rapidly changing complex public health guidance and striving to make evidence-based information quickly accessible to local populations.

*“There’s no question*,* the need was now. The need was not two years down the road.” (CCT14)*.

A collaborative effort drawing on the practical knowledge of ethnic community members was seen to be necessary for effective communication. Critically, each partner perceived mutual benefits from shared purpose and responsibility. For example, public health professionals believed they “*improved transparency*” and advanced “*credibility*” (CCT01) by working with University of Waterloo pharmacists and, through them, a wider network of experts, “*There is a trust with the University’s research*” (CCT10). In turn, community representatives endowed public health messages with greater “*authenticity*” and “*relevance*” (CCT09). Community members also saw gains in more direct access to information *“it will be more helpful for them [fellow community members] to know right away rather than waiting*. *(CCT10)*. Community representatives found a forum to ask questions and seek assurances about vaccine safety that was perceived as independent of government bias; “*people liked that it wasn’t tied to Waterloo Region or Waterloo Public Health or government services*” (CCT01).

Since strong community engagement and involvement was seen to be essential for identifying and prioritising information gaps, contributing to effective infographic design and dissemination, the largest CCT group was focused both on establishing networks, relationships, trust and dialogue that could drive forward on-the-ground communication. Other subgroups concentrated on rapid creation of knowledge translation materials, using the latest research, COVID-19 policy and guidance to create content that answered community concerns about vaccine safety and suitability while simultaneously tailoring it for dissemination efforts. Participants spoke about how the pandemic crisis energised people to act collaboratively, made them prioritise these activities, and create time and resources to accomplish activities.

*“The pandemic spurred an urgency in people; this is actually really important*,* so we do need to prioritise this.” (CCT07)*.

The pandemic further influenced collaboration by changing participants’ frames of reference for interacting. While all CCT participants were affected by “*high population stress*,* huge constant headlines*,* constant conversations*” those with roles and expertise in healthcare and vaccination had “*never been more in demand*” (CCT12). The often ‘*stressful and overwhelming*’ situation of frontline positions and responding to people who were “*very angry and emotional*” (CCT12) created intense focus in the Product Development and Expert Reference’ Groups; people working across multiple groups noticed the strain. However, the conditions also forged new relationships and strengthened co-operativity, “*A friendship situation developed that was very productive*” (CCT12).

#### Virtual collaboration environment

The pandemic caused unparalleled changes to work practices as social interactions were restricted to online activities. Unlike any previous in-person community engagement work undertaken locally, all meetings became virtual, mediated through online platforms. The virtual environment was seen as both helping and hindering interactions that underpinned community engagement; although meeting attendance during lockdown was limited to people with internet access at home, discussions were considered more convenient to schedule and had no travel barriers. Virtual meetings also circumvented some potential barriers such as getting to, or being in, unfamiliar locations. However, whilst turning off cameras and microphones reduced social inhibition, these actions also generated uncertainty about individuals’ attention and reactions. Participants often referred to online interactions missing a dimension of social contact that helped them gauge others’ interest. It was felt that regular in-person meetings would have assisted with building rapport and establishing relationships.

*“Not being able to build community relationships in person like that’s a huge hit” (CCT04*).

### 2) Evolution of infographic development process

#### Establishing a propitious environment for dialogue and collaboration

Collaboration and engagement were shaped by the process of developing tools to support communication about COVID-19 vaccines. This process itself evolved overtime through continued discourse that increased understanding between CCT partners. The organisation of working groups promoted integration of input from community members and those individuals with ideas and expertise in vaccine delivery services, public health, and primary care. This was underpinned by the School of Pharmacy’s repertoire of skills and experience in patient engagement and medication communication. Despite inviting open contributions, it was noted, “*initially some of the community members may not have felt as open’* (CCT01) to speak or were cautious because “*they don’t know how that information will be used*” (CCT07).

The urgency of pandemic circumstances and knowing *“Everybody wants this [.] it’s just we don’t understand each other”* (CCT13) kept people coming back to discussions. Regular interactions assisted by supportive facilitators who called on individuals in turn, by name and “*were just very open to everyone in the group and made sure that everyone did feel comfortable and welcome to share their own thoughts if they wanted to*” (CCT09) helped establish a “*respectful*” environment. Participants described feeling people were *“open to hearing from each other*” (CCT02) because they were “*safe within the space*” (CCT13). Giving voices equal opportunities to be heard and striving to “*make sure that it’s not one-sided or hierarchical or uneven in a power dynamic*” (CCT02), meant meetings had a ‘*collegial environment’* (CCT13), a feeling that was conducive to sharing ideas and decision-making. Building rapport in this way wasn’t seen as a trivial achievement as.

*“In the beginning*,* sometimes it felt like we are conflicting and fighting over having different agendas and so on. That has changed a lot. It did take some time and trust building and meetings and talking [.] building trust can be a very difficult process.” (CCT13)*.

Breaking down barriers, establishing an open, cross-disciplinary discussion forum and common purpose was vital for creating both a positive community involvement experience and constructive outcomes.

*“I’ve come to appreciate the workings of Canada*,* my new country [.] When there’s a will*,* there’s a way.”(CCT05)*.

#### Valuing engagement and adopting community feedback

Community feedback on factual evidence-based information materials was appreciated for “*coming from a place of such authenticity*” and being “*on the ground*” (CCT09). Community spokespersons were careful to voice what they felt was the prevailing discourse in their networks and represented “*what I hear - people’s realities*” (CCT03). One person recalled a conversation where the group ‘*had started to get ahead of ourselves*” (CCT07) and was given a reality check by a community member. The group had been discussing COVID-19 boosters and the community representative reminded them that many people in her community were yet to be fully vaccinated with the primary series.

*“She’d have to ground us again and say*,* ‘We’ve gone too far. We’re not there with you yet*,* bring it back.’ Thinking through those moments*,* especially having her voice in those meetings frequently was really important.” (CCT07)*.

Community involvement was valued for “*keeping us honest*” (CCT12) and the collaborative effort to identify the most relevant topics was considered essential for producing the most meaningful content for use in “*real-time*”.

*“We cannot predict it and think*,* ‘This is what fits in’. Therefore*,* including those voiceless folks into the conversation we can deliver better product.” (CCT06)*.

The collaboration had its challenges though; getting feedback from community representatives took time “*like a slow burn*”, while the expert group sensed “*policy was changing rapidly and we had to be constantly pivoting*,* constantly moving”* (CCT12). Though cycles of reviews and feedback slowed the release of transformative information tools, “*hearing from the people we’re hoping to affect*” and “*honing*” materials to be “*as useful as possible*” (CCT02) was highly valued. Crucially, community members felt their contributions mattered and had positive impact on the final products.

*“I was very privileged to be part of such an awesome team that really challenged themselves*,* to listen to each other and then figure out a way to make things happen.” (CCT06)*.

#### Community-led tailoring of content and dissemination

Involving community representatives was felt to strengthen the quality of infographics: thoughtful input to the group and specific feedback based on practical wisdom about how content and appearance would be received helped improve end products. Over time, the process for integrating feedback evolved from consultation “*Do you have concerns or questions or recommendations before it’s shared out*?” to “*hearing what messages needed to be included and to the actual design*” (CCT11). Community input optimised language, tone, and approach, without it, there was a feeling an infographic might not “*launch*,* get out there or be controversial*” (CCT12). By delivering much more than just small adjustments, community feedback enabled better matching of content to target audiences’ knowledge and lived experiences. Combined with input from frontline vaccinators, community representatives “*were able to bring in*,* ‘This is what they’re asking*’” (CCT12) conceptualizing grassroots concerns. These details improved content and tailoring to better tackle pertinent issues, e.g., decisions regarding vaccine brand as second doses or children’s vaccines.

Adopting feedback wasn’t always straightforward; some topics, such as answering concerns about COVID-19 vaccine effects on fertility, required careful navigation. Developing infographics about COVID infection, vaccination, fertility and child development required balancing appropriate culturally-sensitive and gender-sensitive representations of reproductive health issues. One community representative raised concerns about their peers finding the content too “*vulgar*” and considering public health had a “*hidden agenda to spoil our beliefs*” (CCT05). Another person recalled there was “*a lot of pushback on using gender-sensitive language*” (CCT09) so the group “*tried to strike that balance and still be mindful and true to the community that we were targeting*” (CCT09). A third CCT participant observed navigating this “*fraught space*” required a “*willingness to keep trying in spite of controversy*” and development of a “*brave enough to do it*” attitude (CCT12).

Overall, the partnership between the interdisciplinary, university-based CCT team, public health and community was commended for being “*nimble*,* able to hear feedback and incorporate it*” (CCT11). This was felt to be “really advantageous” to public health in making communication “*more responsive to what we heard from community*” while still being “*supported by endorsement from the Region*” (CCT11).

Beyond tailoring content, the CCT partnership increasingly contributed knowledge about appropriate mechanisms for disseminating information and message acceptance. Participants’ comments underscored “*you need that direct line to community*” (CCT12) to make impact. As the CCT network strengthened and reached deeper into communities, those local influencers “*who will be most listened to”* (CCT04) were identified more often and connected to public health and vaccine advocates. This improved connection aided two-way communication; this was beneficial because “*it opens up transparency*” (CCT01) and gradually generated avenues for wider, more representative, grassroots up feedback. It helped resolve a criticism heard by public health “*Often, we hear from community members; ‘You’re not sharing with us!*’” (CCT01).

Sharing community co-produced materials to healthcare professionals and “*peers in the community who could be champions”* (CCT11), led to more credible voices asserting vaccination as a way of helping individuals and community. Having a multi-cultural, community-based network was valued for contributing to wider vaccine acceptance.

“*Having people that were Muslims*,* that were experts*,* coming out to verify the safety and the authenticity of the vaccines*,* allayed many people’s fears.”* (CCT05).

Improved community networks also aided cross promotion on social media platforms and opened new channels for health communication e.g. WhatsApp groups. Noting “*some populations are not connected electronically*” (CCT03), the CCT helped identify meaningful alternatives such as local businesses, ethnic radio stations and “*religious leaders in particular communities as a source for disseminating knowledge*” (CCT04). An extensive campaign was undertaken to increase vaccine acceptance including opportunities to ask questions through town halls and outreach activities at community centres, mosques and other local venues identified by community members.

### 3) Wider societal context for long-term engagement

#### Acknowledging context

Although resolving the COVID-19 pandemic brought shared purpose to joint efforts, community participants expressed wariness about some aspects of collaborative work based on experiences of discrimination, racism, harm and injustice. Acknowledging this backdrop of historic harm was important for establishing a way forward;

“*Acknowledging the fact that there is mistrust and acknowledging the validity of that concern was more important than ‘here’s the straight up facts around effectiveness and the infection rate with or without a vaccine’.” (CCT11*).

Negative experiences of inequality also had recent dimensions. Early hesitancy was grounded in poor previous experience of consultation, *“Once the data is collected*,* then the community members are pushed to the side.”*  (CCT05). Another participant voiced her community’s frustration with stop-start cycles of community engagement and how it made her hesitant to join the current conversation, “*it’s not going to spread if it’s a one off and it’s done. The challenge is to maintain this*,* to keep it ongoing*” (CCT03). The difficulty of having “*to start the momentum again if anything comes up”* (CCT05) was seen as a weakness of project work that needed addressing through longer term thinking. There was a strong desire to see “*commitment*” from organizations designed to serve the public and a “*willingness to adapt and change…until something is developed that does work*” (CCT03) for the communities they serve.

As relationships between individuals developed, public health and academic partners came to understand the foundations of communities’ hesitancy to trust government institutions and their advice. There was acknowledgment too of the perceived distance between public health and communities served.

*“We heard that a lot from our Community Group [Trust & Relationships Building Group]. Public health used to be of the community and why is it not of the community? Why is it not in the community? Why is it not made from the community? [.] Why is it removed and comes to consult the public? (CCT12).*

The pandemic required a reformulation of community engagement with new players committed to doing more than consulting the public. This suited community members who had said “*from the beginning*” that “*vaccine mobilization wouldn’t be successful if done only through the public health and the doctors*” (CCT05). The CCT presented this new collaborative working arrangement which *“shared responsibility”* (CCT01) and additionally benefited from pharmacists and physicians’ expert knowledge and frontline experience as well as talent in branding and communication.

*“It’s like such a small thing to do*,* but it has such an enormous chance for impact and change and growth and understanding*,* just communicating with the people that are in your community. It breaks down barriers*,* increases understanding and compassion. It’s only good.”(CCT09)*.

#### A transferable model for community engagement

Gathering a diverse group of people with different skills and experiences was perceived to be pivotal in translating complex information into meaningful infographics and reaching under-served audiences, “*the diversity of your team is what predicts your ability to outreach your community*.” (CCT12). This collaborative effort was important because it bridged the divide between policy makers and those with boots-on-the-ground “*people that make policy decisions and recommendations are not really in touch with what’s happening on the ground*” (CCT02). The multi-cultural nature of the CCT was fundamental in this respect,

*“The willingness changes when you have people of different backgrounds who are coming and making the ask” (CCT01)*.

By dovetailing into existing networks and programs, the collaboration aligned ‘*with higher level priorities*” allowing “*resources to be harnessed*” (CCT13); it also provided flexibility, evolving effectively through changing pandemic circumstances and to new community requirements. The CCT approach brought learning opportunities for regional partners to explore new ways of engaging communities, “*that very fluid approach is not something we, as an organization, are used to*” (CCT11).

Interview data revealed there was significant appetite to continue and expand what was seen as improved community engagement that changed how public health acted, “*They listened hard and took it to hear what the community was saying [.] and changed how they do things*” (CCT14). However, continuing engagement was seen as facing several potent challenges including funding and agreeing which priorities to focus on.

*“The long-term goal would be that these relationships are permanent*,* and they’re not only focused on crisis management or a specific issue” (CCT11).*

Other hitches, such as remunerating volunteers, demonstrated how organisational policy needs to be amended to facilitate processes that support how volunteers’ contributions are valued. One contributor noted community volunteers made the same commitments to meetings as public health and university professionals but were not paid equally, nor had equal access to training opportunities or jobs as a result.

Overall, the potential to transfer the CCT model to other scenarios was commended by interview participants “*It’s applicable beyond public health [.] it’s a model that could work in so many different contexts engaging with the public. This is about public facing mobilization*” (CCT04).

## Discussion

Some of the barriers and facilitators to intra-team cooperation described by CCT participants, like the necessity of sharing ideas solely online through virtual meetings and conversations, lie within the context of the pandemic, others speak to experiences of community engagement and collaboration that offer broader insights and learning. Undoubtedly, the COVID-19 pandemic was a potent stimulus for urgent collaborative action to alleviate the social and economic effects of public health measures instigated to reduce infection rates, morbidity and mortality. Academic-community partnerships are well placed to identify and respond to community needs; one mechanism is through sharing accurate, tailored scientific knowledge and education materials [24, 31]. Here, the intentional design of the CCT provided innovation by directly connecting, for the first time, public health professionals with representatives from ethnic minority communities and professionals with expertise in pharmacy, primary care, vaccine delivery, and digital communication to respond to community’s need for timely evidence-based information. Changing public health guidance, complex COVID-19 vaccine policies, circulating misinformation and disinformation and different degrees of local vaccine confidence and literacy, paired with inadequate culturally adapted communication contributed to variable vaccine uptake in Waterloo Region [32]. The intent of CCT participants was to counteract the fear, anxiety, stress, frustration, and confusion arising from this situation, referred to by the WHO as an infodemic [33].

Although the urgency of responding to pandemic circumstances created a driver for collaborative action, urgency can be detrimental to democratic thinking and inclusive action [34]. The dichotomy between the slow pace of collaborative work and the urge to be responsive was mitigated here by ensuring representatives of ethnic minority groups with low vaccination rates were included in sensitive, respectful, open dialogue. By ensuring community-based contributions were heard and incorporated into information resources created, the practical wisdom of grassroots organisations was secured. Creating tailored multimedia information tools was both an objective of the CCT and a vehicle for community involvement that progressed from consultation to fuller community engagement. Accelerating this process by developing community engagement partnerships in the ‘preparedness’, rather than ‘response’ or ‘recovery’ phases of crisis management could sharpen responsiveness and strengthen community resilience [31].

The pandemic context also shaped how relationships, foundational to community involvement, became established as social isolation measures forced CCT dialogue into online spaces. Our data mirrors others’ who have described that while virtual meetings may be convenient, they can be corrupted by unreliable internet connections, domestic disturbances, and privacy interruptions making participants ready to disconnect from purposeful work [35–38]. Virtual meetings feel remote making rapport and relationships difficult to establish, particularly impromptu exchanges between new acquaintances [38]. Here, efforts made to personalize and facilitate equitable contributions created an environment where CCT participants felt valued and prepared for shared decision-making. Making space to acknowledge difficulties, share experiences, support and coping strategies became a transferable feature of dealing with pandemic-related stress that also nurtured relationships and facilitated deeper involvement and engagement. Ghaye suggests empathy, social awareness and attunement are critical features of effective collective, participatory working [39]. A study from California similarly noted co-created restorative healing circles and regular check-ins helped process pandemic stress [24].

Accepting communities hold practical wisdom, resources and capabilities helped academic and public health partners share responsibility for COVID-19 vaccine communication. Familiarity with iterative cycles of consultation on tailoring vaccine information tools that led to improved products helped bi-directional trust evolve. Shared dialogue and consultation transformed into co-production, a core community engagement activity that aligns with progression through IAP2 model stages and holds true to the guiding principles of public engagement in patient-oriented research [40]. Community partners became more involved in monitoring real-time opinion and bringing culturally sensitive nuanced insight to infographic production and dissemination. Repeated use of community input led to more specialized products, translations, and adaptations of language; content better able to serve information needs identified by community representatives. The suite of evidence-based COVID-vaccine related resources created were shared widely in Waterloo Region, Ontario and nationally.

Experiencing mutual benefit through CCT interactions kept people returning to discussions, collaborative effort and began sustained co-operation. Communities identified as ‘difficult-to-reach’ were known to be exposed to structural inequalities in provision of health and social services such as language barriers in the Region of Waterloo. Acknowledging problems like historic harm and systemic racism, were important steps towards a successful coalition and levelling local power hierarchies. A commitment to work with racialized, minority ethnic or other disadvantaged groups and critically, act alongside trusted community representatives, promoted public health’s credibility. It started to address the criticism of not being “of the community, for the community” and combat “outreach fatigue”, the response to top-down interventions that don’t address community identified needs [41].

Sustained co-operation and partnership with local, trusted leaders are known to broker trust, enhance audience receptivity and lend authenticity to institutions’ communication [24–26, 31, 42, 43]. In our scenario, communities with links to Somali, Nigeria, Rohingya and Ethiopia were among those who sought and gained better information about and access to COVID-19 vaccines through participation in the CCT, and via its extended networks. The time invested in developing mutual understanding benefited these communities as they became empowered to arrange their own vaccination clinics according to their preferences. In ceding control to community voices, the highest level of IAP2 engagement, public health benefited from better utilisation of local resources and recruitment of new community actors able and willing to address vaccine hesitancy and promote COVID-19 vaccine uptake. Longer-term strategies of community engagement reflect more authentic investment that transforms questionable interventions into tailored, trustworthy collective good [21, 25, 41, 44].

Community volunteers in CCT provided valuable, critical insights about their roles that should inform future engagement work. A discrepancy in remuneration and access to opportunities for future employment was experienced between volunteers and professionals involved in the CCT. The contributions of active citizens and community-based organisations in public health responses need to be recognised and valued, den Broeder argues this will enable and sustain resilient and confident ‘disaster proof’ communities [45]. Whether in calm or crisis mode, volunteers’ discretionary efforts contribute significantly to successful service delivery. To be sustainable, collaborations need to inspect how they support volunteers’ abilities and willingness to contribute making structural and systemic changes if needed.

Acknowledgement of systemic racism and new co-operation helped bridge the gap between public health & community. Over-time, and through commitment, relationships were formed around shared goals and responsibility. There was strong endorsement for this approach and interest in continuing this form of open dialogue to further advance advocacy and action around other social injustice issues. It takes time to change attitudes, but community advocates are powerful catalysts of change, resourceful and knowledgeable about how to achieve it. Furthermore, the CCT model is transferable to other community engagement opportunities. The value of bringing in a range of backgrounds and experience enabled contributions from experts in their field, frontline practitioners, operational coordinators and communities combine to tackle big issues. Importantly, it represents a mechanism for grassroots feedback to those who design systems and make policy. The positive lessons learned about multidisciplinary collaboration and community engagement situated within an urgent pandemic landscape can be harnessed and applied to co-designed strategy for and delivery of healthcare provision, research and translated into systematic community engagement in wider fields.

### Limitations

This study is based on a collaboration in Waterloo Region around the development of vaccine communication tools. As described, the pandemic circumstances fostered increased interest in and commitment to a collaborative project focused on identifying and responding to emergent community information needs. As such, the work, and subsequent interview data, was biased towards the needs of groups with representation; groups that didn’t participate remain unheard voices. Furthermore, qualitative data were drawn from interviews with CCT participants, a necessarily limited pool that may not reflect the views of all contributors to wider outreach discussions. Social desirability bias was also a potential limitation of this study as participants reflected on activities that they had contributed to and therefore recalled in favourable light.

The relative contributions of authors make a methodological consideration of this study; two CCT participants (MT, and NW) who contributed to interview data also reviewed the findings of the thematic analysis and three interview participants reviewed the draft manuscript (MT, NW, KG). We mitigated potential biases arising by focusing on the analysis and interpretations of data by researchers who were independent of the CCT process.

## Conclusion

A multidisciplinary group from public health, pharmacy, primary care, and community representatives (CCT) collaborated on the production of information tools to advance COVID-19 vaccine confidence and acceptance among underserved ethnic minority populations in the Region of Waterloo. Community involvement deepened to fuller engagement through an extended process of open, respectful dialogue and iterative feedback on content, design and dissemination of information tools. Sustained, collegial co-operation, growing mutual understanding and appreciation of others’ needs, and experiences together with repeated, demonstratable use of community feedback helped bridge the ‘otherness’ of public health messages to underserved, equity-deserving communities.

## Electronic supplementary material

Below is the link to the electronic supplementary material.

Supplementary Material 1

## Data Availability

Reasonable requests for access to original data can be made to N Waite.

## Abbreviations

PHAC: Public Health Agency Canada
CCT: Connect Collaborate Tailor
