# Supplementary material for: Connect, collaborate and tailor: a model of community engagement through infographic design during the COVID-19 pandemic
Source: BMC Public Health. 2024 Sep 19;24:2551. doi: 10.1186/s12889-024-20037-3 (PMC11411729; doi:10.1186/s12889-024-20037-3)
Supplement: Supplementary file 1 — Supplementary Material 1 [file 12889_2024_20037_MOESM1_ESM.docx]

**Appendix 1**

IAP2 Framework for community engagement (29)


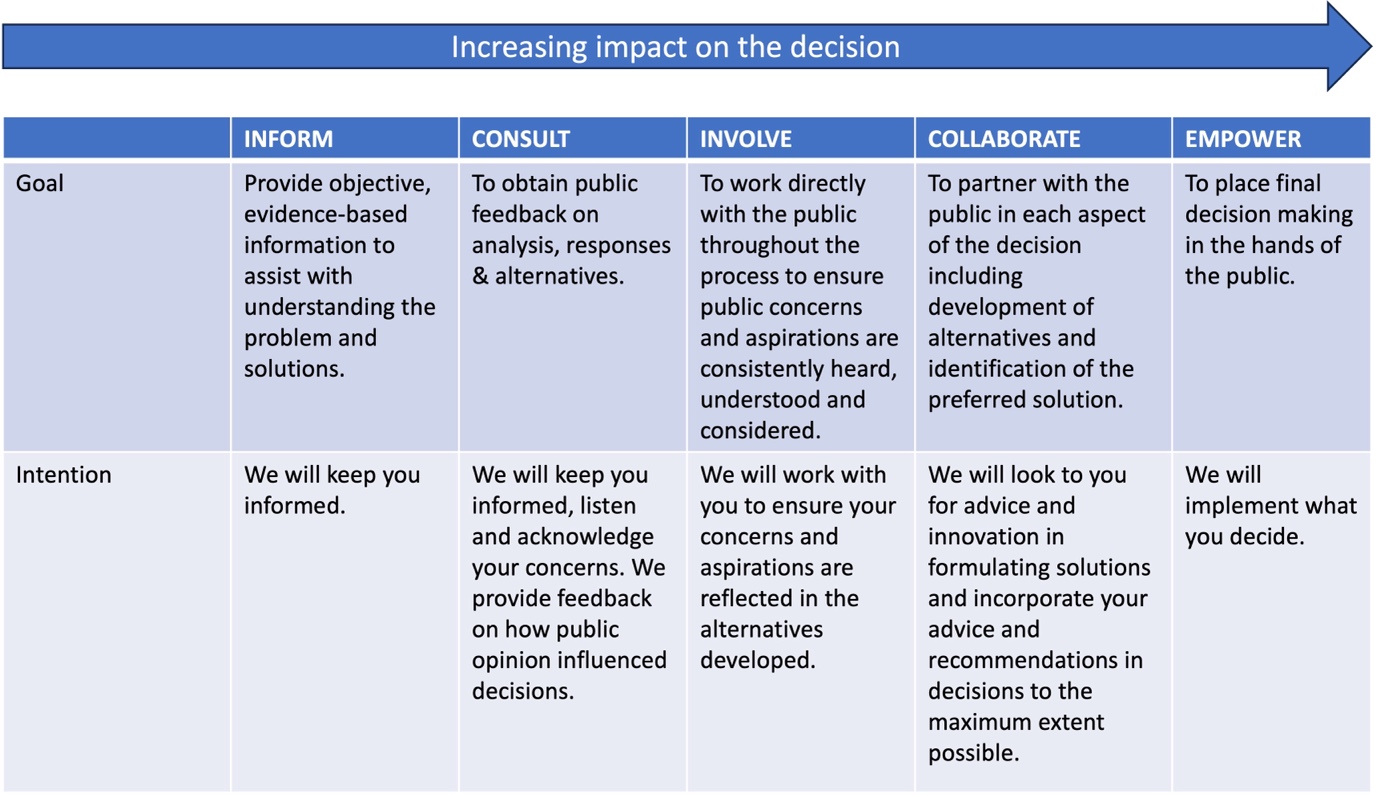


**Appendix 2**

Roles and Affiliations of CCT participants who contributed to evaluation interviews.

| **CCT working group** | **Partnership affiliations/professional role of participants who participated in interviews** |
| --- | --- |
| Relationship & Trust Building Group | Region of Waterloo public health, (Director & junior manager infectious disease control, nursing); health promotion analyst  Family physician  City of Kitchener community engagement facilitator  Community representatives; Anti-racism Advisory Working Group, Waterloo Region School Board, Mayor’s Advisory Council for Kitchener Seniors, Peace for All Canada, African Women’s Alliance of Waterloo  University of Waterloo academics from the School of Pharmacy, Department of Communication Arts, Waterloo Centre for Microbial Research |
| Product Development Group | University of Waterloo, School of Pharmacy  Public health/ family physician |
| Media Engagement Group | University of Waterloo; School of Pharmacy, Department of Communication Arts, |
| Research Group | University of Waterloo; School of Pharmacy |
| Expert Reference Group | Primary care physicians and hospitalists; Researchers in infectious diseases and vaccines at McMaster, University of Toronto, Western, Laval Universities. |

**Appendix 3**

**CCT evaluation interview questions**

Intro script: Thank you for finding the time to meet with me. The research team is conducting interviews to collect your feedback on the Connect-Collaborate-Tailor project (we will call this CCT during the interview). More specifically, we would like to understand your perspective and experience with community connections and the development of the COVID-19 infographics.

1. What do you think the benefits are of community-engaged and co-designed projects in the health promotion and prevention space?

- Why do you think this is an important approach to take in the vaccine confidence space?

1. Can you tell me about the role you played in the community engagement part of the CCT process?
2. How did community engagement and involvement change over time? Can you give me examples?
3. Was the CCT process effective in identifying and addressing the vaccine confidence related concerns of the community?

- Can you give an example of when this went well?
- Can you give an example of when this could have been done better?
- Are there other community members who should have been involved or engagement strategies you think should have been used in this work?

1. How did community engagement impact the infographic content, language, or other end-product design?

- Can you give examples of infographics where you saw this occur?

1. How did the process of making the infographic change over time?

- Why did these changes occur?
- Are there some key lessons learned that could be used earlier on in infographic development in other projects?
- What were some of the challenges that still remained in the infographic development process?

1. How did the uptake of the infographics change over time (who was using them, where they were used, how they were used)?

- Can you give some examples?

1. Do you think the infographics produced met the needs of the community, if so, why do you think they were successful, or if not, why do you think they didn’t meet the needs?

- Can you give examples where you saw this occur?

What were some of the measures you considered in determining whether the infographic met the community’s needs?

1. How did the infographics impact the community’s perspectives about COVID-19?

- How did you know whether the infographic did or did not have impact? Can you provide examples?

1. What were the most challenging aspects of CCT process?

- How were these challenges overcome? (example: managing conflict, not enough/effective engagement with community, scientific community or public health)

1. What were some of the key benefits for public health achieved by this project? For your organization (if you are not in public health)?

- Do you think these benefits are sustainable for similar future work?
- Were there any additional public health benefits that you hoped would occur but did not? If so, what were the potential barriers to achieving these benefits?

1. What were some of the key barriers to doing community engagement/infographic development at your institution?
2. How is public health/your organization better equipped to address vaccine hesitancy in the community after participating in this project?
3. Can you tell me as an individual and as an organization, what did you learn about trust, relationships and the importance of working together (community, public health, scientific community, public health, researchers) through your involvement in this project?
4. This project was about engaging community and meeting vaccine information needs during a pandemic, so things happened fast, vaccine information needs changed quickly, and communities were in a state of flux as they were impacted by and adapted to the pandemic.

- What were the positive and negative aspects of doing our community engagement and infographic development work in this environment?

1. What aspects of this project would you like to continue, or would you use again, in other projects/activities?

- What types of resources would you need to be able to do this?

1. Is there anything else you would like to say about this project, your involvement, or its impact before we close the interview?

**Community Members Interview Guide:**

Intro script: Thank you for finding the time to meet with me. The research team is conducting interviews to collect your feedback on the Connect-Collaborate-Tailor project (we will call this CCT during the interview). More specifically, we would like to understand your perspective and experience making community connections and the development of the COVID-19 infographics.

1. Why was it important for you to be involved in this project and support efforts to increase vaccine confidence in your community?
2. Can you tell me about the role you played in this project?

- Is there an example you can share of when what you brought to the project was important and/or made a difference?
- How do you think your role was viewed by others in this project?
- How did your role change over time?

1. What did you learn about collaborating with public health and/or researchers after being involved in this project?

- How do you think we could better encourage other community members to engage in a project like this in the future?

1. What difficulties or challenges did you experience while participating in this project?

- How did you or others overcome these?
- How do you think we could improve the way we engaged with you and your community?

1. Was this project effective at capturing and addressing the priority vaccine concerns of your community and/or the communities served by this project?

- Can you give examples of when this did and did not occur?
- Were there any other community members you would have like invited or other ways of involving the community in this project?

1. As you and/or your community worked on this project did you feel your opinions were listened to?

- Did you/your community have enough opportunities to provide your opinion?
- Do you feel you/your community’s opinions were impactful on the work that was done?

1. Can you see you/your community opinions reflected in the final infographics?

- Can you give examples in terms of infographic topics chosen, information content, language used or design?
- Did the final products use language/content that made them accessible, appropriate and culturally safe for you/your community?

1. Are there examples of infographics that still didn’t meet the needs of you/your community? Please explain further.

- Do you feel the infographics supported people in the community to make their own decisions about vaccination?
- Can you give an example?
- How did the infographics impact how community members talked about vaccines?

1. What would you say are some of the key benefits achieved by involving communities like yours in this work?

- prompts: listened to, involvement, centring community voices, accessible resources, meaningful information, empowerment, self-determination, etc…]
- How is this community better equipped to address vaccine hesitancy after this project is finished?
- What do you think the remaining challenges are?

1. How can we increase trust, foster collaboration, and strengthen relationships between the community and public health/researchers/science experts?

- Why do you think it is important that we strengthen trusts and relationships with respect to vaccine hesitancy?

1. This project was about engaging community and meeting vaccine information needs during a pandemic, meaning things happened fast, vaccine information needs changed quickly, and communities were in a state of flux as they were impacted by and adapted to the pandemic.

- What were the positive and negative aspects of doing our community engagement and infographic development work in this environment?

1. What aspects of this project would you like to continue, or would you use again in other projects/activities?

- What types of resources would you need to be able to do this?

1. Is there anything else you would like to say about this project, your involvement, or its impact before we close the interview?
